# Joint mapping of genes and conditions via multidimensional unfolding analysis

**DOI:** 10.1186/1471-2105-8-181

**Published:** 2007-06-05

**Authors:** Katrijn Van Deun, Kathleen Marchal, Willem J Heiser, Kristof Engelen, Iven Van Mechelen

**Affiliations:** 1 SymBioSys, Catholic University of Leuven, 3000 Leuven, Belgium; 2 Department of Microbial and Molecular Systems, Catholic University of Leuven, 3000 Leuven, Belgium; 3 Department of Psychology, Leiden University, 2300 RB Leiden, The Netherlands

## Abstract

**Background:**

Microarray compendia profile the expression of genes in a number of experimental conditions. Such data compendia are useful not only to group genes and conditions based on their similarity in overall expression over profiles but also to gain information on more subtle relations between genes and conditions. Getting a clear visual overview of all these patterns in a single easy-to-grasp representation is a useful preliminary analysis step: We propose to use for this purpose an advanced exploratory method, called multidimensional unfolding.

**Results:**

We present a novel algorithm for multidimensional unfolding that overcomes both general problems and problems that are specific for the analysis of gene expression data sets. Applying the algorithm to two publicly available microarray compendia illustrates its power as a tool for exploratory data analysis: The unfolding analysis of a first data set resulted in a two-dimensional representation which clearly reveals temporal regulation patterns for the genes and a meaningful structure for the time points, while the analysis of a second data set showed the algorithm's ability to go beyond a mere identification of those genes that discriminate between different patient or tissue types.

**Conclusion:**

Multidimensional unfolding offers a useful tool for preliminary explorations of microarray data: By relying on an easy-to-grasp low-dimensional geometric framework, relations among genes, among conditions and between genes and conditions are simultaneously represented in an accessible way which may reveal interesting patterns in the data. An additional advantage of the method is that it can be applied to the raw data without necessitating the choice of suitable genewise transformations of the data.

## Background

Complex microarray experiments profile the expression of a large number of genes under different conditions (environmental conditions, knockout experiments, patients), and/or over time. Depending on the biological question at hand, one may be interested in finding subsets of genes that can be clustered together based on similarities in their overall expression profile, or in finding subsets of conditions (tissues, patients) that can be grouped together based on similarities in their overall gene profile. Also more subtle relations between genes and conditions can be envisaged, such as biclusters of genes being co-expressed over a subset of conditions only (modules) or groups of genes being discriminative for subsets of conditions. However, the massive amount of information and relations present in the data, pose a challenge for the data analyst: It is not trivial to know where to start looking for structure and a priori choices can have the consequence that something is missed. For instance, many cluster algorithms require defining in advance the number of clusters to be searched for, a parameter which is difficult to guess in advance. Therefore, having a rough idea on the most prominent patterns present in the data and the (unexpected) particularities, prior to performing a more profound analysis may be most useful. Exploratory methods offer the possibility to reduce the data to a manageable amount of information, for example by means of a clustering of the individual elements to a small number of groups or by means of reducing them to a small number of dimensions (e.g., PCA/SVD). Often, such methods yield insightful graphical representations. Ideally, such representations should display genes and conditions *jointly *in a way that associations amongst genes, amongst conditions and between genes and conditions are all three easy to grasp.

From this perspective, multidimensional unfolding (MDU) seems a promising data exploration technique (for an introduction to MDU see [1] and [2]): This method maps both genes and conditions into the same low-dimensional space such that, 1) genes are located closest to the conditions for which they exhibit the highest expression levels, 2) genes (respectively conditions) with a more similar expression profile are located closer to each other in the space. The resulting MDU configurations are very easy to interpret and give a quick first insight into the overall structure of the data and its particularities. An additional asset of the method, is that it can be applied to raw gene expression data: In contrast to results obtained from other exploratory methods, results of MDU are independent of gene-specific transformations applied to the input data.

Although theoretically suitable as a data exploration technique, current MDU algorithms cannot readily be applied due to problems of a general kind and of problems that are specific for the case of microarray gene expression data. As regards problems of a general kind: first, some algorithms do not converge to a local minimum and yield unstable results; second, in many cases MDU representations are not well interpretable due to a sticking together of a majority of gene and condition points implying that they cannot be discriminated from one another. As regards problems that are specific for the case of gene expression data, first, existing MDU algorithms have not been designed for the analysis of data sets of the typical sizes of microarray data as they require a large amount of memory; second, existing MDU algorithms also are computationally very intensive (e.g., because they rely on matrix inversions). To deal with these problems, in the present paper we propose a novel MDU algorithm. A subsequent application of it to two publicly available microarray datasets, each of which serving a different biological purpose, will demonstrate its exploratory power.

## Results

### Method

The purpose of a multidimensional unfolding of gene expression data is to find coordinates in a low-dimensional space, both for the genes and the conditions, in a way that the (Euclidean) distance of a gene point to a condition point is shorter the higher the gene is expressed in that condition. Note that MDU can be considered as an extension of multidimensional scaling (MDS) to the rectangular case (see chapter 14 of [1]). To formalize MDU we will use the following notation: Genes are indexed by *i *= 1 ... *n*, the conditions by *j *= 1 ... *m*, and the dimensions of the low-dimensional space by *r *= 1 ... *p*. Also, let **E **be the *n *× *m *expression matrix for the *n *genes measured in *m *conditions with *e*_*ij *_representing the expression of gene *i *in condition *j *and **e**_*i *_the *m*-sized vector representing the expression profile of gene *i*.

To map **E **to a *p*-dimensional space, a *n *× *p *matrix of gene coordinates **X **and a *m *× *p *matrix of condition coordinates **Y **are sought such that the Euclidean distances for gene *i*, contained in the *m*-sized vector **d**_*i *_and with dij=[∑r=1p(xir−yjr)2]0.5
 MathType@MTEF@5@5@+=feaafiart1ev1aaatCvAUfKttLearuWrP9MDH5MBPbIqV92AaeXatLxBI9gBaebbnrfifHhDYfgasaacH8akY=wiFfYdH8Gipec8Eeeu0xXdbba9frFj0=OqFfea0dXdd9vqai=hGuQ8kuc9pgc9s8qqaq=dirpe0xb9q8qiLsFr0=vr0=vr0dc8meaabaqaciaacaGaaeqabaqabeGadaaakeaacqWGKbazdaWgaaWcbaGaemyAaKMaemOAaOgabeaakiabg2da9iabcUfaBnaaqadabaGaeiikaGIaemiEaG3aaSbaaSqaaiabdMgaPjabdkhaYbqabaGccqGHsislcqWG5bqEdaWgaaWcbaGaemOAaOMaemOCaihabeaakiabcMcaPmaaCaaaleqabaGaeGOmaidaaOGaeiyxa01aaWbaaSqabeaacqaIWaamcqGGUaGlcqaI1aqnaaaabaGaemOCaiNaeyypa0JaeGymaedabaGaemiCaahaniabggHiLdaaaa@4AE5@, reflect the expression profile **e**_*i *_of gene *i*.

To find **X **and **Y **such that *n *vectors of distances **d**_*i *_reflect the expression profiles **e**_*i*_, we will maximize

1n∑i=1,r(ei,di)<0nr2(ei,di),
 MathType@MTEF@5@5@+=feaafiart1ev1aaatCvAUfKttLearuWrP9MDH5MBPbIqV92AaeXatLxBI9gBaebbnrfifHhDYfgasaacH8akY=wiFfYdH8Gipec8Eeeu0xXdbba9frFj0=OqFfea0dXdd9vqai=hGuQ8kuc9pgc9s8qqaq=dirpe0xb9q8qiLsFr0=vr0=vr0dc8meaabaqaciaacaGaaeqabaqabeGadaaakeaadaWcaaqaaiabigdaXaqaaiabd6gaUbaadaaeWbqaaiabdkhaYnaaCaaaleqabaGaeGOmaidaaOGaeiikaGccbeGae8xzau2aaSbaaSqaaiabdMgaPbqabaGccqGGSaalcqWFKbazdaWgaaWcbaGaemyAaKgabeaakiabcMcaPaWcbaqbaeqabiqaaaqaaiabdMgaPjabg2da9iabigdaXiabcYcaSaqaaiabdkhaYjabcIcaOiab=vgaLnaaBaaameaacqWGPbqAaeqaaSGaeiilaWIae8hzaq2aaSbaaWqaaiabdMgaPbqabaWccqGGPaqkcqGH8aapcqaIWaamaaaabaGaemOBa4ganiabggHiLdGccqGGSaalaaa@4E83@

the average squared correlation between the expression profiles and the distances in the low-dimensional representation. Because higher expression levels are to correspond to shorter distances, the summation runs only over those genes for which there is a negative correlation between the expression levels and the Euclidean distances (denoted by *r*(**e**_*i*_, **d**_*i*_) < 0). In order to maximize (1), the coordinate matrices **X **and **Y **have to be such that the distance vectors correlate as negatively as possible with the profiles while positive correlations are to be avoided. An important aspect of the optimization criterion, is that any set of positive *genewise *linear transformations of the raw expression data **E**, will yield the same optimal **X**, **Y**, because the correlation is insensitive to linear transformations; as such, tough questions about preprocessing, insofar they pertain to gene-specific linear transformations are bypassed.

### Algorithm

To find **X **and **Y **that maximize (1), we reformulate this optimization problem to an equivalent one, namely minimizing

1n∑i=1nvar⁡(eistd(ei)+aidi),
 MathType@MTEF@5@5@+=feaafiart1ev1aaatCvAUfKttLearuWrP9MDH5MBPbIqV92AaeXatLxBI9gBaebbnrfifHhDYfgasaacH8akY=wiFfYdH8Gipec8Eeeu0xXdbba9frFj0=OqFfea0dXdd9vqai=hGuQ8kuc9pgc9s8qqaq=dirpe0xb9q8qiLsFr0=vr0=vr0dc8meaabaqaciaacaGaaeqabaqabeGadaaakeaadaWcaaqaaiabigdaXaqaaiabd6gaUbaadaaeWbqaaiGbcAha2jabcggaHjabckhaYnaabmaabaWaaSaaaeaaieqacqWFLbqzdaWgaaWcbaGaemyAaKgabeaaaOqaaiabbohaZjabbsha0jabbsgaKjabcIcaOiab=vgaLnaaBaaaleaacqWGPbqAaeqaaOGaeiykaKcaaiabgUcaRiabdggaHnaaBaaaleaacqWGPbqAaeqaaOGae8hzaq2aaSbaaSqaaiabdMgaPbqabaaakiaawIcacaGLPaaaaSqaaiabdMgaPjabg2da9iabigdaXaqaaiabd6gaUbqdcqGHris5aOGaeiilaWcaaa@4EFB@

(with var and std denoting variance and standard deviation respectively) with respect to *a*_*i*_, **X**, and **Y **under the constraint that *a*_*i *_≥ 0 for all *i*; see the appendix for a proof of the equivalence. For reasons given below, two more constraints are added to the optimization problem: First, the *a*_*i *_weights are bounded by an upper bound *u *(such that 0 ≤ *a*_*i *_≤ *u*); and second, ||**x**_*i*_|| ≤ 1 for all *i *in a space centered at the point of gravity for the condition coordinates **y**_*j*_. Note that centering can be done without loss of generality.

For the minimization of (2) with respect to the *a*_*i*_'s, **X**, and **Y **under the constraints 0 ≤ *a*_*i *_≤ *u *and ||**x**_*i*_|| ≤ 1, we propose the algorithm GENEFOLD (which may be considered a major upgrade of the algorithm proposed in [3]). A detailed description of it along with a MATLAB implementation can be found at [4]. GENEFOLD is of an alternating least squares type. In each iteration the *a*_*i*_'s, **X**, and **Y **are updated each in turn while the remaining parameters are kept fixed. The (constrained) update of the *a*_*i *_weights can be done on the basis of a closed form expression (see appendix). The update of the gene coordinates **X **under the constraint ||**x**_*i*_|| ≤ 1 for all *i*, as well as the update of the condition coordinates **Y**, are based on a numerical technique called iterative majorization (see [5-7] for the use of iterative majorization in the context of multidimensional data analysis). Briefly said, iterative majorization relies on surrogate objective functions with the following properties: The surrogate function is easier to minimize than the original, it lies above the original function, and the surrogate function touches the original function in the so-called supporting point. By choosing the supporting point equal to the minimum of the surrogate function in the previous iteration, the sequence of loss-values will be non-increasing.

GENEFOLD solves both the general MDU problems and the problems that are specific for gene expression data. With respect to the general problems, first convergence is guaranteed because the proposed algorithm yields a non-increasing sequence of loss values for a function which is bounded below (by zero). Second, the problem of a lack of discrimination of the coordinates such that a majority of points stick together (which is known as the degeneracy problem in MDU), is solved by the constraints *a*_*i *_≤ *u *and ||**x**_*i*_|| ≤ 1. 1) Due to the restriction ||**x**_*i*_|| ≤ 1 in a space centered at the point of gravity of the condition coordinates, the gene points lie on the unit sphere, and 2) limiting *a*_*i *_to values smaller than or equal to *u *with the value of *u *well chosen (see the appendix), pulls the variance of the distances **d**_*i *_to the variance of the distances on the unit sphere with uniformly distributed points. With regard to problems that are specific for the analysis of (large) gene expression data sets; first, GENEFOLD works on considerably smaller matrices than the (*n *+ *m*) × (*n *+ *m*) used in classical procedures for MDU (in which MDU is treated as a special case of MDS); second, GENEFOLD does not rely on computationally intensive methods like matrix inverses [1,8]. As an illustration of the computational speed of GENEFOLD: with 100 iterations, the analysis of a 517 × 12 matrix takes about a second and of a 6075 × 173 matrix about 6 minutes (on a desktop, Pentium 2.80 GHz4 CPU with 0.99 GB RAM).

### Applications

We applied multidimensional unfolding to two publicly available data sets, one situated in an experimental context [9] where the study aimed at characterizing the temporal program of gene expression in human fibroblasts and one situated in a clinical setting [10] where the aim was to classify two tissue types on the basis of the gene expression levels.

#### Time-course gene expression data

The data discussed in [9] pertain to the temporal change of genes in human fibroblasts that had been deprived from serum for 48 hours which causes them to enter a nondividing state. The deprivation was ended by addition of a medium containing fetal bovine serum (FBS) and micro-array hybridization was performed at several moments during the 24 hours following serum stimulation. We will analyze the 517 genes that were also retained by [9] and that can be obtained at [11].

**Figure 1:**
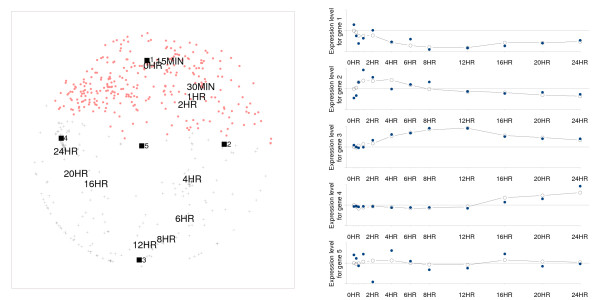
**Unfolding configuration for time experiment data**. Unfolding configuration (left panel) and derived expression profiles (right panel) for five selected genes. Genes labeled by a dot show a repression peak, while genes labeled by a cross show an induction peak compared to the initial time point 0 hr. Some genes are labeled by a black square and also have a numbered label: their derived expression profile is given in the right panel (the connected black dots), together with the original profile (the unconnected blue dots).

Because our unfolding algorithm relies on an iterative procedure with a non-convex solution space and a prespecified dimensionality, some consideration has to be given to the choice of a stopping rule, to the problem of local minima, and to the dimensionality of the configuration. With respect to the stopping rule, we chose to terminate the iterative procedure when the difference in loss between the current and previous solution was less than 10^-5^; our experience with this value is that it yields stable solutions (in the sense that more iterations result in almost the same configuration and loss) in a reasonable amount of time. The problem of local minima was accounted for by restarting the algorithm 101 times, using 100 semi-rational starts and a rational start for the initial coordinates, the solution with the lowest loss being retained. The dimensionality of the configuration is determined by a comparison of loss values: For the one up to five-dimensional solution, loss was respectively 0.55, 0.25, 0.19, 0.15, 0.11, which suggests a two-dimensional configuration (one dimension less results in a considerable increase in loss while more dimensions barely reduce the loss). For the two-dimensional configuration, a very good fit was obtained: The average genewise correlation between the distances and the raw data is -0.86. A visual representation of the solution is depicted in Figure 1 where the genes are denoted by dots and the time points by self-explanatory labels.

A striking feature of Figure 1 is the clocklike organization of the time points, which is characterized by the following features: First, the points lie approximately on an elongated circle; second, they are ordered according to time; and third, the last time points fold somewhat back to the earliest. Note that no information on the order of the time points is included in the unfolding analysis; the ordered outcome is therefore not a trivial finding. The unfolding analysis also reveals that there is little differentiation between some time points; for example, the time points 0 hr and 15 min are clustered together, which means that expression 15 minutes after stimulation is barely different from expression during the nondividing state (0 hr); the same holds for the time points 30 min, 1 hr, 2 hr and 16 hr, 20 hr, 24 hr, while the time points 4 hr, 6 hr, 8 hr, 12 hr are more spread out. The large gaps between 2 hr and 4 hr and between 12 hr and 16 hr suggest a biological event occurring within these time intervals.

Taking a look at the genes, we see that these, too, are organized in a circular way with a blank spot in the middle. Another feature to look at, is the location of the majority of the genes: Most are located close to the earliest and latest time points, whereas only a few genes are located at intermediate time points. The expression of a gene at the different time points is reflected by the distances from this gene to the time points: The closer a time point is located to a gene, the higher the expression level or, conversely, the more distant a time point is from a gene, the lower the expression level. Note that for these data, we know that the expression at 0 hr corresponds to a neutral state, such that higher expression levels indicate induction and lower expression levels repression. This means that induction occurs at time points close by while repression occurs at distant time points. For ease of interpretation, we used distinctive labels for genes with an induction peak and with a repression peak: Genes with an induction peak being those for which the difference in distance between the reference time point and the time point closest to the gene point is larger then the difference in distance between the reference time point and the time point furthest from the gene point (i.e., genes for which the largest difference in expression level from the expression level at 0 hr is positive, respectively negative). Remember further that the distances between gene and condition points inversely reflect the expression level. Consider, for example, the gene represented by the square numbered one in Figure 1 (this is close to 0 hr): From the unfolding configuration, it can be derived that this gene will have its highest expression at 0 hr, the time point that is closest to it; continuing in a time-wise direction, expression decreases up to 12 hr as is reflected by the increasing distance; from that point on, the distances become progressively shorter, which suggests that the expression levels steadily increase. The resulting expression profile is plotted in the upper part of the right panel of Figure 1 (the connected dots represent the profile as modeled by the distances; the non-connected dots the original data profile); the horizontal axis represents time, the vertical axis the expression levels as they are modeled by the unfolding representation. The time axis is proportional to real time (e.g., the tick mark for 12 hr is placed 48 times further than the tick mark for 15 min); the modeled expression levels are obtained (per gene) by subtracting the distance from the distance at time 0 hr and multiplying these distances by minus one. In the expression profiles in Figure 1, the neutral state is indicated by the horizontal (reference) line. Thus the configuration suggests that gene one, and all those that are close to it, will be repressed soon after stimulation with the highest repression occurring from 8 to 12 hours. Therefore, for genes close to gene one, there appears to be little or no induction. Otherwise, the gene labeled one (gene number 21 in the original data set), belongs to the first cluster of genes found by [9] and is an inhibitor of the progression of the cell-cycle division (p57 Kip2). Profiles for other genes can be derived in a similar way: In Figure 1, four additional profiles are given for the genes numbered two to five in the left panel. Note that the gene numbered five, which is located in the center of the clock, badly fits the original data: The corresponding derived profile is irregular and unstable in that small changes in the location of this gene would result in another profile, given the fact that all time points are almost equally distant.

Taguchi and Oono [12] analyzed the same data, leaving out the preset expression level at time 0 hr. They applied nonmetric multidimensional scaling on the matrix of dissimilarities between genes where dissimilarities were measured by the Pearson correlation coefficient with the sign flipped. As a result they obtained a two-dimensional configuration in which the genes were arranged on the edge of a circular structure. To detect the temporal regulation, the authors subsequently drew the configuration at each time point, plotting only those genes that exceeded a preset expression peak: As shown in [12], the expression peaks move gradually around the circle in a timely fashion. These authors also take up the discussion on the periodicity of genes in relation to the cell-cycle. They argue that the ring-like structure is in favor of periodicity in the data. Yet, undoubtedly, the unfolding approach is a much better technique to tackle this substantive issue: In case of periodicity, the time points will fall approximately on a circle with time points that are separated *k *periods falling together. As illustrated by Figure 1, the clockwise organization of the time points suggests some periodicity but the fact that the last time points do not coincide with the earliest ones, does not fit within a periodic frame. Given the experimental difficulties encountered in studies that involve temporal regulation of genes, we do not wish to draw any conclusions concerning the presence or absence of periodicity in this particular data set. It should be clear, however, that multidimensional unfolding is a particularly suitable analysis technique to deal with such an issue.

#### Colon cancer data

Many applications of gene expression profiling can be found in clinical settings where genome-wide expression is measured for different patient or tissue groups. A challenge for the exploratory MDU tool within this setting may be to retrieve useful information beyond a mere identification of genes that optimally discriminate between the different groups.

We consider gene expression data for 62 colon tissues, 40 of which are tumorous (colon adenocarcinoma) and 22 normal [10] (see [13] for the data in MATLAB format). To obtain those genes that discriminate optimally between the groups, we selected of the 2000 available genes those 400 that have the highest correlation with the binary classifier (normal versus tumor). The 400 × 62 expression matrix was subjected to a MDU analysis yielding the following loss values for the one up to five dimensional solutions: 0.80, 0.38, 0.31, 0.27, and 0.24. The two-dimensional solution offered the best trade off between fit and sparseness with an average correlation between distances and expression profiles amounting to -0.78.

##### Tissues

**Figure 2:**
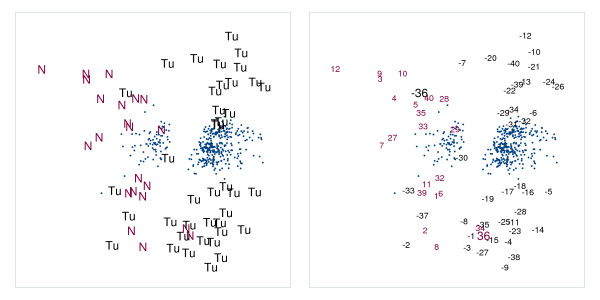
**Unfolding configuration for colon cancer data: Normal versus tumor**. Unfolding configuration for the colon cancer data: The left and right panels only differ in the labels used for the tissues. Genes are labeled by blue dots in both panels; tissues in the left panel are labeled with 'N' for the normal tissues and with 'Tu' for the tumor tissues while in the right panel they are labeled in function of the patient number with positive numbers indicating a normal tissue for that patient and negative numbers a tumor tissue.

**Figure 3:**
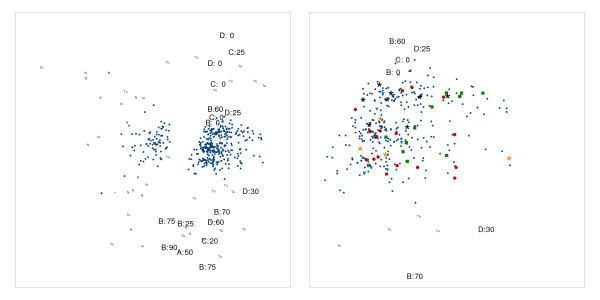
**Unfolding configuration for colon cancer data: Two tumor types**. Unfolding configuration for the colon cancer data: In the left panel, tissues are labeled for the normal tissues with 'N' and for the tumor tissues either with 'Tu' or a label indicating both the Duke stage and the percentage of contamination with normal tissue. In the right panel, a detail of the unfolding configuration is given that zooms in on the region containing genes that are more highly expressed in the tumor tissues. Different colors and symbols are used to discern the different functional gene groups: Red dots for the ribonucleoprotein genes, green squares for the ribosomal protein genes, blue triangles for the proteasome genes, orange asterisks for genes involved in protein folding, and black asterisks for protein kinase genes.

As to be expected, the normal and colon cancer tissues are separated in the MDU configuration, see the left panel of Figure 2: At the left, we find the normal tissues (labeled by N) and at the right the tumorous ones (labeled by Tu); two normal tissues are erroneously grouped with the tumors and, conversely, five tumor samples are placed with the normal samples (these results are comparable with the results in [10]). However, the right panel of Figure 2 in which the tissues are labeled according to the patient number (such that the same number corresponds to the same patient) includes a clear indication that in case of patient number 36 the tissues have been mislabeled rather than misclassified. A further aspect of the MDU configuration that jumps to the eye, is the separation of the cancer tissues in two groups, one located in the upper right of Figure 2 and one in the lower right. We cannot be certain of the cause of this separation, but based on the available patient descriptions, it might be related to the contamination of tumor tissue with normal tissue, the cancer stage, or both. In Figure 3 some of the tissues are labeled in function of the percentage of contamination with normal tissue, and the stage of the cancer (ranging from A, early stage, to D, metastasis). It can be seen that the top group contains tissues of a more developed cancer stage and are less contaminated than the bottom group (this information was found in [14]). The fact that more contaminated tissues do not shift towards the group of normal tissues, might suggest that the degree of contamination is not the prime cause for the observed subdivision. Related to this, all misclassifications are situated in the region containing the more contaminated tissue samples.

##### Genes

Taking a look at the genes in Figure 2 (left panel), there are two clearly separated groups, one associated to the normal tissues and one associated to the tumor tissues. To retrieve the functional annotation of the genes in these groups, we used DAVID [15] on a selection of genes that are fitted well by the MDU representation (*r*(**e**_*i*_,**d**_*i*_) ≤ -0.80). For the genes closer to the normal tissues, consistent with the results in [10], we found a cluster of genes annotated with muscle contraction. As to the genes associated to the tumor tissues, several functional groups were discerned which are further labeled using different symbols and colors in the right panel of Figure 3. These functional groupings seem to hint at an elevated cellular metabolism reflecting the higher metabolic activity and division rate typical for cancer cells: a group of ribonucleoprotein genes (red dots), a group of ribosomal protein genes (green squares), a group of proteasome genes (blue triangles), a group of protein folding genes (yellow stars), and a group of kinases related to cell cycle regulation.

## Discussion

Multidimensional unfolding can be a useful tool when dealing with the challenging task of extracting useful information from microarray gene expression data: As shown in this paper, MDU yields easy-to-grasp representations and appears to be a versatile tool for data exploration that may reveal many kinds of interesting patterns present in the data. For example, in the first application, an intriguing clock-like structure for the time points was revealed, a pattern that has not been uncovered in a direct way up to present for these well-studied data; in the second application, the unfolding analysis revealed an intriguing subdivision of the cancer tissue groups, beyond a mere discrimination of normal and tumor tissues. A possible limitation of the unfolding approach as presented here, is that in case of a large number of heterogeneous conditions, low-dimensional configurations can be obtained that are mainly blurred due to the actual high-dimensional structure of the data. Also, a huge number of genes can result in a configuration that provides little insight into the data. A possible way to overcome this limitation could be the use of a hybrid approach that results in low-dimensional distance-based representations of clustered data. Such an approach, has already been proposed for multidimensional scaling [16] and for the clustering of row elements in metric multidimensional unfolding [17].

## Appendix

### Equivalence of loss functions

We show the equivalence of minimizing loss function (2) and maximizing (1). Consider the loss function for one gene,

var⁡(eistd(ei)+aidi)=var⁡(ei)var⁡(ei)+2aistd(ei)cov⁡(ei,di)+ai2var⁡(di)=1+2air(ei,di)std(di)+ai2var⁡(di).
 MathType@MTEF@5@5@+=feaafiart1ev1aaatCvAUfKttLearuWrP9MDH5MBPbIqV92AaeXatLxBI9gBaebbnrfifHhDYfgasaacH8akY=wiFfYdH8Gipec8Eeeu0xXdbba9frFj0=OqFfea0dXdd9vqai=hGuQ8kuc9pgc9s8qqaq=dirpe0xb9q8qiLsFr0=vr0=vr0dc8meaabaqaciaacaGaaeqabaqabeGadaaakeaafaqadeGadaaabaGagiODayNaeiyyaeMaeiOCai3aaeWaaeaadaWcaaqaaGqabiab=vgaLnaaBaaaleaacqWGPbqAaeqaaaGcbaGaee4CamNaeeiDaqNaeeizaqMaeiikaGIae8xzau2aaSbaaSqaaiabdMgaPbqabaGccqGGPaqkaaGaey4kaSIaemyyae2aaSbaaSqaaiabdMgaPbqabaGccqWFKbazdaWgaaWcbaGaemyAaKgabeaaaOGaayjkaiaawMcaaaqaaiabg2da9aqaamaalaaabaGagiODayNaeiyyaeMaeiOCaiNaeiikaGIae8xzau2aaSbaaSqaaiabdMgaPbqabaGccqGGPaqkaeaacyGG2bGDcqGGHbqycqGGYbGCcqGGOaakcqWFLbqzdaWgaaWcbaGaemyAaKgabeaakiabcMcaPaaacqGHRaWkcqaIYaGmdaWcaaqaaiabdggaHnaaBaaaleaacqWGPbqAaeqaaaGcbaGaee4CamNaeeiDaqNaeeizaqMaeiikaGIae8xzau2aaSbaaSqaaiabdMgaPbqabaGccqGGPaqkaaGagi4yamMaei4Ba8MaeiODayNaeiikaGIae8xzau2aaSbaaSqaaiabdMgaPbqabaGccqGGSaalcqWFKbazdaWgaaWcbaGaemyAaKgabeaakiabcMcaPiabgUcaRiabdggaHnaaDaaaleaacqWGPbqAaeaacqaIYaGmaaGccyGG2bGDcqGGHbqycqGGYbGCcqGGOaakcqWFKbazdaWgaaWcbaGaemyAaKgabeaakiabcMcaPaqaaaqaaiabg2da9aqaaiabigdaXiabgUcaRiabikdaYiabdggaHnaaBaaaleaacqWGPbqAaeqaaOGaemOCaiNaeiikaGIae8xzau2aaSbaaSqaaiabdMgaPbqabaGccqGGSaalcqWFKbazdaWgaaWcbaGaemyAaKgabeaakiabcMcaPiabbohaZjabbsha0jabbsgaKjabcIcaOiab=rgaKnaaBaaaleaacqWGPbqAaeqaaOGaeiykaKIaey4kaSIaemyyae2aa0baaSqaaiabdMgaPbqaaiabikdaYaaakiGbcAha2jabcggaHjabckhaYjabcIcaOiab=rgaKnaaBaaaleaacqWGPbqAaeqaaOGaeiykaKIaeiOla4caaaaa@A62E@

Equation (3) is a plain quadratic form in *a*_*i *_which, under the constraint *a*_*i *_≥ 0, reaches its minimum at

ai=−r(ei,di)std(di),
 MathType@MTEF@5@5@+=feaafiart1ev1aaatCvAUfKttLearuWrP9MDH5MBPbIqV92AaeXatLxBI9gBaebbnrfifHhDYfgasaacH8akY=wiFfYdH8Gipec8Eeeu0xXdbba9frFj0=OqFfea0dXdd9vqai=hGuQ8kuc9pgc9s8qqaq=dirpe0xb9q8qiLsFr0=vr0=vr0dc8meaabaqaciaacaGaaeqabaqabeGadaaakeaacqWGHbqydaWgaaWcbaGaemyAaKgabeaakiabg2da9maalaaabaGaeyOeI0IaemOCaiNaeiikaGccbeGae8xzau2aaSbaaSqaaiabdMgaPbqabaGccqGGSaalcqWFKbazdaWgaaWcbaGaemyAaKgabeaakiabcMcaPaqaaiabbohaZjabbsha0jabbsgaKjabcIcaOiab=rgaKnaaBaaaleaacqWGPbqAaeqaaOGaeiykaKcaaiabcYcaSaaa@44ED@

if *r*(**e**_*i*_, **d**_*i*_) < 0, and at 0 if *r*(**e**_*i*_, **d**_*i*_) ≥ 0. Substituting the optimal *a*_*i*_'s in loss function (2) yields

1−1n∑r(ei,di)<0r(ei,di)2.
 MathType@MTEF@5@5@+=feaafiart1ev1aaatCvAUfKttLearuWrP9MDH5MBPbIqV92AaeXatLxBI9gBaebbnrfifHhDYfgasaacH8akY=wiFfYdH8Gipec8Eeeu0xXdbba9frFj0=OqFfea0dXdd9vqai=hGuQ8kuc9pgc9s8qqaq=dirpe0xb9q8qiLsFr0=vr0=vr0dc8meaabaqaciaacaGaaeqabaqabeGadaaakeaacqaIXaqmcqGHsisldaWcaaqaaiabigdaXaqaaiabd6gaUbaadaaeqbqaaiabdkhaYjabcIcaOGqabiab=vgaLnaaBaaaleaacqWGPbqAaeqaaOGaeiilaWIae8hzaq2aaSbaaSqaaiabdMgaPbqabaGccqGGPaqkdaahaaWcbeqaaiabikdaYaaaaeaacqWGYbGCcqGGOaakcqWFLbqzdaWgaaadbaGaemyAaKgabeaaliabcYcaSiab=rgaKnaaBaaameaacqWGPbqAaeqaaSGaeiykaKIaeyipaWJaeGimaadabeqdcqGHris5aOGaeiOla4caaa@4A8D@

Obviously minimizing (5) is equivalent to maximizing (1).

### Choice upper bound on *a*_*i*_'s and constrained update

From Equation (4), it can be seen that subjecting the *a*_*i*_s to an upper bound *u *attracts the solution space to configurations with a positive lower bound on std(**d**_*i*_), the spread of the distances, and this will be the more so the stronger the distances correlate with the expression profiles. A suitable value for *u *depends on the range of the coordinates: We propose to work in a reference space centered at the point of gravity of the condition coordinates and with ||**x**_*i*_|| ≤ 1 (such that the gene coordinates lie within the unit sphere).

For this reference space, the upper bound for *a*_*i *_is set equal to *u *= (*mv*)^-0.5 ^with *v *the variance of the Euclidean distance from a point *i *to points sampled uniformly in the unit sphere of dimensionality *p *with *v *calculated using Monte Carlo simulation. Using this upper bound for *a*_*i *_will, for a maximal (absolute) correlation *r*(**e**_*i*_, **d**_*i*_) = -1, pull the variance of the distances towards *v *or larger values. The constrained update for *a*_*i *_becomes then

ai={0,if−r(ei,di)std(di)≤0,−r(ei,di)std(di),if 0<−r(ei,di)std(di)<u,u,if−r(ei,di)std(di)≥u.
 MathType@MTEF@5@5@+=feaafiart1ev1aaatCvAUfKttLearuWrP9MDH5MBPbIqV92AaeXatLxBI9gBaebbnrfifHhDYfgasaacH8akY=wiFfYdH8Gipec8Eeeu0xXdbba9frFj0=OqFfea0dXdd9vqai=hGuQ8kuc9pgc9s8qqaq=dirpe0xb9q8qiLsFr0=vr0=vr0dc8meaabaqaciaacaGaaeqabaqabeGadaaakeaacqWGHbqydaWgaaWcbaGaemyAaKgabeaakiabg2da9maaceqabaqbaeaabmGaaaqaaiabicdaWiabcYcaSaqaaiabbMgaPjabbAgaMjabgkHiTmaalaaabaGaemOCaiNaeiikaGccbeGae8xzau2aaSbaaSqaaiabdMgaPbqabaGccqGGSaalcqWFKbazdaWgaaWcbaGaemyAaKgabeaakiabcMcaPaqaaiabbohaZjabbsha0jabbsgaKjabcIcaOiab=rgaKnaaBaaaleaacqWGPbqAaeqaaOGaeiykaKcaaiabgsMiJkabicdaWiabcYcaSaqaaiabgkHiTmaalaaabaGaemOCaiNaeiikaGIae8xzau2aaSbaaSqaaiabdMgaPbqabaGccqGGSaalcqWFKbazdaWgaaWcbaGaemyAaKgabeaakiabcMcaPaqaaiabbohaZjabbsha0jabbsgaKjabcIcaOiab=rgaKnaaBaaaleaacqWGPbqAaeqaaOGaeiykaKcaaiabcYcaSaqaaiabbMgaPjabbAgaMjabbccaGiabicdaWiabgYda8iabgkHiTmaalaaabaGaemOCaiNaeiikaGIae8xzau2aaSbaaSqaaiabdMgaPbqabaGccqGGSaalcqWFKbazdaWgaaWcbaGaemyAaKgabeaakiabcMcaPaqaaiabbohaZjabbsha0jabbsgaKjabcIcaOiab=rgaKnaaBaaaleaacqWGPbqAaeqaaOGaeiykaKcaaiabgYda8iabdwha1jabcYcaSaqaaiabdwha1jabcYcaSaqaaiabbMgaPjabbAgaMjabgkHiTmaalaaabaGaemOCaiNaeiikaGIae8xzau2aaSbaaSqaaiabdMgaPbqabaGccqGGSaalcqWFKbazdaWgaaWcbaGaemyAaKgabeaakiabcMcaPaqaaiabbohaZjabbsha0jabbsgaKjabcIcaOiab=rgaKnaaBaaaleaacqWGPbqAaeqaaOGaeiykaKcaaiabgwMiZkabdwha1jabc6caUaaaaiaawUhaaaaa@9A4E@

## Authors' contributions

KVD developed the algorithm and applied it to the two gene expression data sets discussed. IVM, KM, and KVD drafted the manuscript. KM and KE made substantial contributions to the biological background. WJH and IVM scrutinized the unfolding method and its application to gene expression data. All authors read and approved the final manuscript.
